# Systematic protein interactome analysis of glycosaminoglycans revealed YcbS as a novel bacterial virulence factor

**DOI:** 10.1038/srep28425

**Published:** 2016-06-21

**Authors:** Felix Shih-Hsiang Hsiao, FX Reymond Sutandy, Guan-Da Syu, Yi-Wen Chen, Jun-Mu Lin, Chien-Sheng Chen

**Affiliations:** 1 Graduate Institute of Systems Biology and Bioinformatics, National Central University, Jongli District, Taoyuan City 32001, Taiwan; 2 Department of Biomedical Science and Engineering, National Central University, Jongli District, Taoyuan City 32001, Taiwan

## Abstract

Microbial pathogens have evolved several strategies for interacting with host cell components, such as glycosaminoglycans (GAGs). Some microbial proteins involved in host–GAG binding have been described; however, a systematic study on microbial proteome–mammalian GAG interactions has not been conducted. Here, we used *Escherichia coli* proteome chips to probe four typical mammalian GAGs, heparin, heparan sulphate (HS), chondroitin sulphate B (CSB), and chondroitin sulphate C (CSC), and identified 185 heparin-, 62 HS-, 98 CSB-, and 101 CSC-interacting proteins. Bioinformatics analyses revealed the unique functions of heparin- and HS-specific interacting proteins in glycine, serine, and threonine metabolism. Among all the GAG-interacting proteins, three were outer membrane proteins (MbhA, YcbS, and YmgH). Invasion assays confirmed that mutant *E. coli* lacking *ycbS* could not invade the epithelial cells. Introducing plasmid carrying *ycbS* complemented the invading defects at *ycbS* lacking *E. coli* mutant, that can be further improved by overexpressing *ycbS*. Preblocking epithelial cells with YcbS reduced the percentage of *E. coli* invasions. Moreover, we observed that whole components of the *ycb* operon were crucial for invasion. The displacement assay revealed that YcbS binds to the laminin-binding site of heparin and might affect the host extracellular matrix structure by displacing heparin from laminin.

The extracellular matrix (ECM) is a major structure comprising molecules that facilitate cell adhesion, cell-to-cell communication, and differentiation among various animal cells^1^. The ECM components are intracellularly produced by resident cells; once secreted, these molecules deposit and subsequently assemble into the existing matrix^2^. The ECM is composed of a laminated mesh of fibrous proteins and glycosaminoglycans (GAGs)^3^,^4^,^5^,^6^. GAGs are carbohydrate polymers that comprise variably sulphated disaccharide repeats of β4GlcUA–α4GlcNAc or β4GlcUA–β3GalNAc. The β4GlcUA–α4GlcNAc repeats contribute to the synthesis of heparin and heparan sulphate (HS), whereas the β4GlcUA–β3GalNAc repeats contribute to the synthesis of chondroitin sulphate B (CSB) and chondroitin sulphate C (CSC)^7^. GAG chains, including HS, CSB, and CSC, are commonly attached to core proteins on the ECM, forming negatively charged proteoglycans. By contrast, heparin is secreted from degranulated mast cells and exists as a free GAG chain, which interacts with the cell surface^8^. These GAGs exhibit diverse cellular functions according to their composition and polysaccharide chain size^7^,^9^.

Several studies have reported that GAGs are involved in cellular structure, recognition, adhesion, and signalling^4^,^10^,^11^. In addition, GAGs interact with microbial proteins, and these interactions are substantial for microbial pathogenicity^6^,^12^. Some pathogens have evolved GAG-binding proteins (GBPs) that recognise host GAGs and use the advantages of these molecules^13^,^14^. For example, such binding facilitates bacterial attachment to the host cell surface and in some cases even mediates pathogen dissemination^14^. Bacterial adhesins are the most common type of bacterial virulence factors and facilitate the adherence of bacterial cells to the host surface. Many fimbrial-type adhesins recognise GAGs and thus mediate attachment to the host as a critical step in the pathogenesis of most infections^15^.

Some microbial virulence factors involved in host–GAG binding have been described; however, a systematic study on microbial proteome and mammalian GAG interactions has not been reported. Protein microarrays have recently emerged as a useful and powerful tool for conducting high-throughput protein interaction studies. Several proteome microarrays have been fabricated, such as yeast^16^, *Escherichia coli*^17^, *Arabidopsis*^18^, and human proteome microarrays^19^,^20^. These studies have recently been applied to various research areas, including protein–protein, protein–lipid, protein–DNA, protein–peptide, protein–cell, and protein–small molecule interactions^21^. Here, we conducted a proteome-wide screening of the GAG interactome by using *E. coli* proteome chips containing approximately 4300 nonredundant proteins. We used four typical mammalian GAGs, namely heparin, HS, CSB, and CSC, to profile the interactions of these molecules with the *E. coli* proteome. Several intracellular and membrane-bound GBPs were identified after thorough screening and bioinformatics analysis. We validated three outer membrane GBPs: MbhA, YcbS, and YmgH. YcbS was confirmed as a major molecule for bacterial infection.

## Results

### Identification of the GAG-interacting proteome by using *E. coli* proteome chips

We performed proteome-wide screening of the GAG interactome by using *E. coli* proteome chips. The combined system approaches were used to assess novel GBPs, and the key virulence factors were accordingly determined. As shown in Supplementary Fig. S1, we expressed and purified approximately 4300 *E. coli* proteins to fabricate the *E. coli* whole proteome chip. These proteins were purified using a high-throughput protein purification protocol and subsequently printed onto chips in duplicate^17^. We used four typical mammalian GAGs labelled with DyLight 650, heparin, HS, CSB, and CSC to profile the interactions of these molecules with the *E. coli* proteome. The GBPs were identified using a local cutoff of the mean + 2 standard deviations (SDs), which refers to 2 SDs higher than the regional signal mean (protein spot area, 9 × 9) of the specific protein spot. Bioinformatics analyses, namely Kyoto Encyclopedia of Genes and Genomes (KEGG) pathway mapping and motif searches, were performed to determine the biological relevance of the identified GBPs. The outer membrane GBPs were subsequently identified, and their functions were validated using *E. coli* invasion assays involving knockout, complementation, overexpression, and blocking strategies. The GAG–GBP interactions were further confirmed using binding affinity (Kd) assays and through flow cytometry. Moreover, the action of GBP hits on GAGs was evaluated using displacement assays.

**Figure 1:**
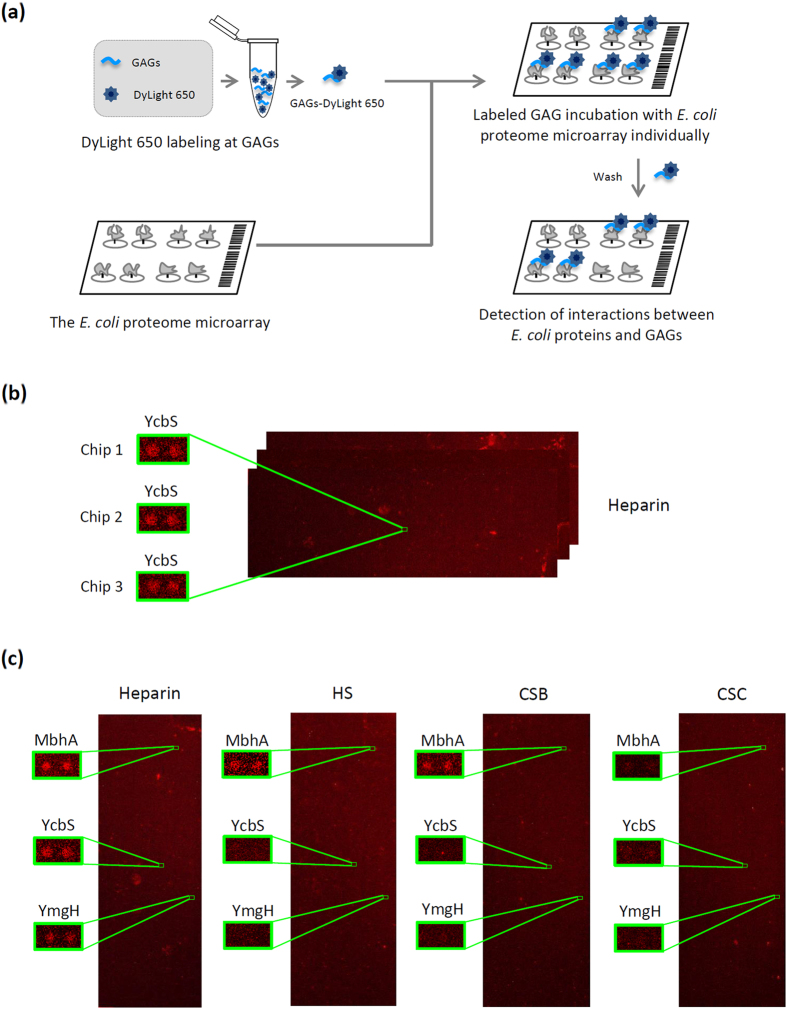
Schematic diagram of *E. coli* proteome chip assays. (**a**) *E. coli* proteome chips probed with heparin, HS, CSB, and CSC. The GAGs were labelled with DyLight 650 (GAGs-DyLight 650) and individually probed using the *E. coli* proteome chips. After washing to remove excess unbound GAGs, the chips were scanned to detect the binding signals. (**b**) To determine the reproducibility of the chip assay, the chip image of YcbS and heparin from triplicate experiments are shown. (**c**) The three GAG-binding protein signals MbhA, YcbS, and YmgH are outlined using a rectangle in the probing results for heparin, HS, CSB, and CSC.

In the chip assay, we chemically labelled the GAGs with DyLight 650 N-hydroxysuccinimide (NHS) ester (Fig. 1a), which reacted with the primary or secondary amines to generate stable linkages. The DyLight 650-labeled GAGs were individually incubated with the *E. coli* proteome chip. The representative binding protein signals are shown in Fig. 1b. We used a cutoff value of mean + 2 SDs and identified 185, 62, 98, and 101 proteins as GBPs for heparin, HS, CSB, and CSC, respectively (Supplementary Data S1). No binding signal was identified from the negative control image (only DyLight 650 without GAG).

### KEGG pathway analysis of the identified GBPs

We used KEGG to delineate the biological relevance of the identified GBPs. Among all the GBPs, two enriched functions (lipopolysaccharide biosynthesis and ribosome) were identified. N-acetylglucosamine (GlcNAc)-harbouring heparin and HS were reported to regulate virulence properties in microbes^22^; therefore, we further analysed the KEGG function in heparin- and HS-specific GBPs, which bound to heparin or HS but not to CSB or CSC. Overall, 105 heparin- and HS-specific GBPs exist. The results revealed enhanced lipopolysaccharide biosynthesis in heparin- and HS-specific GBPs. In addition, among these GBPs, we identified a unique enriched function governing glycine, serine, and threonine metabolism, suggesting that many heparin- and HS-specific GBPs function in bacterial amino acid metabolism. Amino acid metabolism involves virulence factor synthesis in bacteria^23^; therefore, these findings have further clarified the biological roles of heparin- and HS-specific GBPs in regulating amino acid metabolism, thereby potentially regulating bacterial virulence factor synthesis.

### Motif search of heparin- and HS-specific GBPs

We used GLAM2 to identify all the known motifs occurring in a sequence of heparin- and HS-specific GBPs. Specifically, we identified a unique consensus sequence, XPAX(0,1)EA[SV]X(0,1)EXQ[LV]X(0,2)[AR]RLLR, for this GBP group (Supplementary Fig. S2). In addition, the residue 13–18 (X(0,2)[AR]RLLR) of this motif matched five of the six previously reported known binding motifs of heparin^24^. This motif likely represents the binding site of heparin.

Bioinformatics analyses revealed that the membrane-bound GBPs possibly yielded the most intriguing observation. Because of the cellular location of these molecules, the outer membrane-bound GBPs have a higher likelihood of establishing direct contact with the host-cell GAGs and might thus contribute to bacterial attachment and invasion. Gene ontology of the cellular component of the GBPs by using the chip assays yielded three outer membrane proteins: MbhA, YcbS, and YmgH. MbhA binds to heparin, HS, and CSB. YcbS and YmgH bind to heparin alone (Fig. 1c). These three GBPs were subsequently used in further analyses to examine the roles of these molecules in host–pathogen interactions.

### YcbS is crucial for *E. coli* invasion into host cells

**Figure 2:**
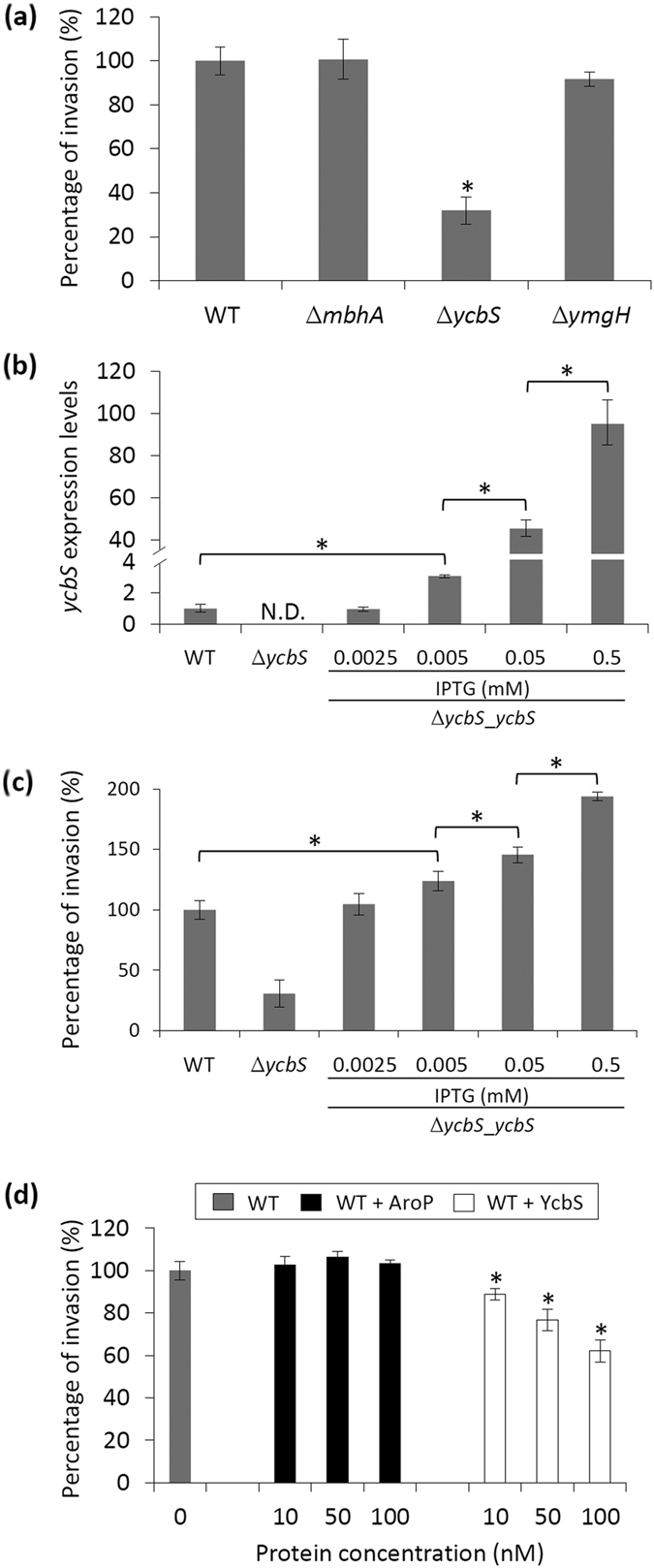
*E. coli* invasion assays of outer membrane GBPs. (**a**) The Keio *E. coli* mutants lacking a single GBP gene were subjected to invasion assays. The *ycbS*-lacking *E. coli* mutant (∆*ycbS*) showed significantly lower invasion than did the WT control. (**b**) *ycbS* mutants (∆*ycbS*) were transformed with the plasmid pCA24N–*ycbS* to form ∆*ycbS***_***ycbS*; QPCR analysis showed that YcbS in ∆*ycbS* can be induced to yield a WT level of expression with 0.0025 mM IPTG and further overexpression with IPTG in a dose-dependent manner. The pCA24N plasmid without a gene insertion was used as a negative control vector in WT and ∆*ycbS*. N.D. represents a nondetectable value. The asterisk represents a statistically significant difference in *ycbS* expression by the *E. coli* in each group (*p* < 0.05). Data are shown as the mean of three biological replicates. (**c**) The invasion assay revealed that expressed *ycbS* complemented the invading defects of ∆*ycbS* when the bacteria were treated with 0.0025 mM IPTG. Further inducing *ycbS* plasmids with an increasing concentration of IPTG dose-dependently enhanced the percentage of successful bacterial invasions of the ∆*ycbS*_*ycbS* strain (*p* < 0.05). Invaded bacteria were counted and standardised as percentages. Controls were set to 100%. The asterisks represent statistically significant differences in the invasion efficiency of *E. coli* in each group (*p* < 0.05). The data are shown as the mean of five biological replicates. (**d**) The binding of purified YcbS to the HCT-8 cell surface gradually reduced the *E. coli* invasion of the host cells in addition to the increase in YcbS (10, 50, and 100 nM), as demonstrated using blocking assays, compared with the WT control. The AroP-treated cells (negative control) showed no blocking effects. Invaded bacteria were counted and standardised as percentages. Controls were set to 100%. The asterisks represent statistically significant difference in the invasion efficiency of *E. coli* compared with that of the WT control (*p* < 0.05). The data are shown as the mean of five biological replicates.

We investigated whether the binding of the identified outer membrane proteins is critical for the invasion of *E. coli* into host cells. The three outer membrane proteins were knocked down to investigate the relevance of these molecules in bacterial infections by using invasion assays (Supplementary Fig. S3). Specifically, the bacteria were added to the human ileocecal epithelial cell line HCT-8 for invasion. The extracellular noninvading bacteria were killed after incubating the HCT-8 cells with gentamycin for 1 h^25^. The cytosol-invading bacteria were subsequently released after disrupting the HCT-8 cells with 0.1% Triton X-100 and quantified through the spread plate method. A wild-type (WT) control was used as a comparison to calculate the percentage of bacterial invasion. As shown in Fig. 2a, only *ycbS*-lacking *E. coli* mutants (∆*ycbS*) showed a significant decrease in bacterial invasion compared with the WT control (*p* < 0.05). We subsequently transformed the *ycbS* plasmid (pCA24N–*ycbS*) to *∆ycbS*. The YcbS in ∆*ycbS* was induced to yield a WT level of expression with 0.0025 mM IPTG. The YcbS can be further induced with higher concentrations of IPTG in a dose-dependent manner. YcbS was nondetectable in ∆*ycbS* (Fig. 2b). The invasion assay showed that compared with the invasion ability of the WT control, that of *∆ycbS* was complemented by the plasmids carrying *ycbS* at 0.0025 mM IPTG induction (Fig. 2c). Moreover, *ycbS* induction at higher concentrations of IPTG enhanced the bacterial invasions in ∆*ycbS*_*ycbS* (*p* < 0.05; Fig. 2c). This result indicates that YcbS directly contributed to the capability of *E. coli* to infect host cells.

Next, we determined whether the outer membrane protein YcbS blocks *E. coli* invasion into the HCT-8 cells. We preincubated the HCT-8 cells with different concentrations (0, 50, and 100 nM) of purified YcbS, followed by assessment by using invasion assays. The AroP protein was used as the negative control because it is not in our GBP list. The results revealed that blocking with YcbS gradually reduced the percentage of successful *E. coli* invasion into the host cells compared with the WT control cells (*p* < 0.05; Fig. 2d). The negative control cells exhibited no blocking effects. This result further confirms that YcbS facilitates *E. coli* invasion.

### The *ycb* operon plays a critical role in *E. coli* invasion into host cells

**Figure 3:**
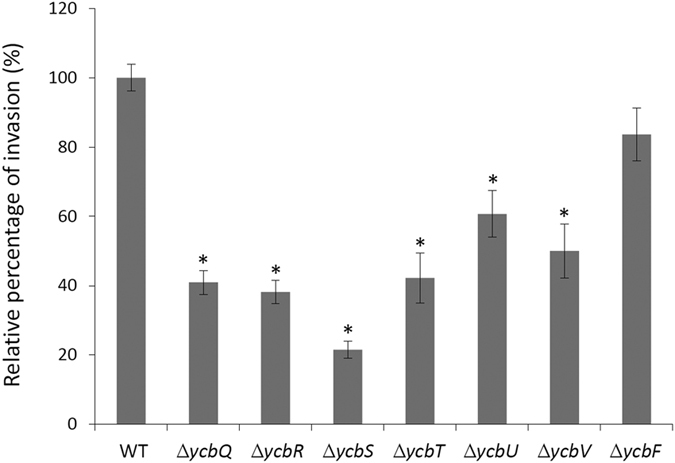
Characterisation of the *ycb* operon proteins by using the *E. coli* invasion assay. Seven *ycb*-lacking *E. coli* mutants were cultured with HCT-8 cells and subjected to invasion assays. Except for *ycbF*, all mutant *ycb* strains showed a lower invasion capacity than did the WT control. Invaded bacteria were counted and standardised as percentages. Controls were set to 100%. The asterisks represent the statistically significant difference in the invasion efficiency of *E. coli* mutant strains compared with that of the WT control (*p* < 0.05). The data are shown as the mean of five biological replicates.

YcbS is a component of a putative chaperone-usher fimbrial operon in *E. coli*^26^. Therefore, we investigated whether the other components of this operon also contribute to *E. coli*–host cell interactions. We performed invasion assays in different *E. coli* mutants lacking *ycbQ*, *ycbR*, *ycbS*, *ycbT*, *ycbU*, *ycbV*, or *ycbF*. To ensure the invasion assay result was solely because of the invasion ability, not the growth ability in the assay period, the *ycb*-lacking *E. coli* mutants were examined to ensure that they exhibited equivalent growth curves with the WT control (Supplementary Fig. S4). Our results showed that all the mutants, except *ycbF*, had a lower invasion ability than the WT control did (*p* < 0.05; Fig. 3), indicating that the *ycb* operon plays a major role in bacterial invasion.

### Binding affinity between YcbS and heparin

**Figure 4:**
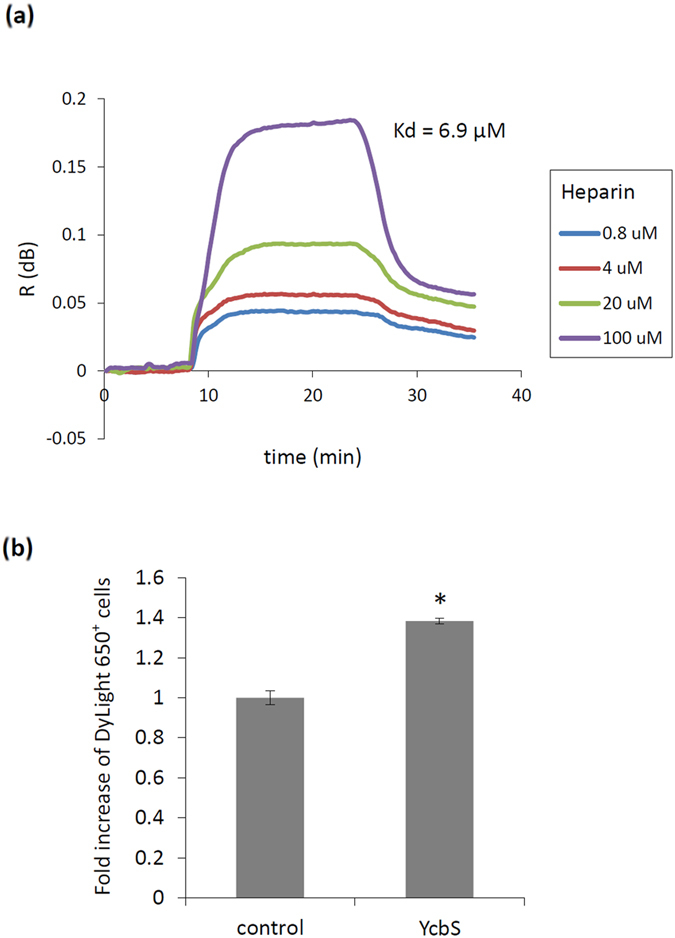
Affinity of the YcbS–heparin interaction and confirmation of the YcbS–cell interaction. (**a**) The binding affinity of heparin to the YcbS protein was determined through SPR interaction analysis. The sensorgram revealed real-time association and dissociation SPR profiles corresponding to heparin concentrations on the immobilised YcbS chip. The calculated Kd of YcbS–heparin binding was 6.9 μM. (**b**) Flow cytometry revealed that YcbS bound to HCT-8 epithelial cells demonstrated significantly higher DyLight 650 binding signals than did the WT control (*p* < 0.05). The bar graph represents the fold increases in the signals increases in the HCT-8 cells incubated with YcbS and DyLight 650-labeled anti-His antibody compared with those incubated with DyLight 650-labeled anti-His antibody alone. The data are shown as the mean of three biological replicates.

YcbS showed a strong binding affinity for heparin (Fig. 1b); therefore, we further measured the Kd of YcbS to heparin by using surface plasmon resonance (SPR). Purified YcbS was first immobilised onto the SPR sensor chip. Different concentrations of heparin (0.8, 4, 20, and 100 μM) were subsequently used to probe with the YcbS chip. The result revealed that YcbS was bound to heparin with a Kd of 6.9 μM (Fig. 4a).

We then investigated whether YcbS binds to the heparinised surfaces of mammalian cells through flow cytometry. HCT-8 cells were also used as a host cell model. A DyLight 650-labeled anti-Histidine (His) antibody was used to stain YcbS. Moreover incubation with the anti-His antibody alone was considered the negative control. DyLight 650 signals on cells incubated with YcbS were significantly higher (*p* < 0.05; Fig. 4b), thus confirming that YcbS binds to HCT-8 cells.

### YcbS displaces heparin–laminin binding

**Figure 5:**
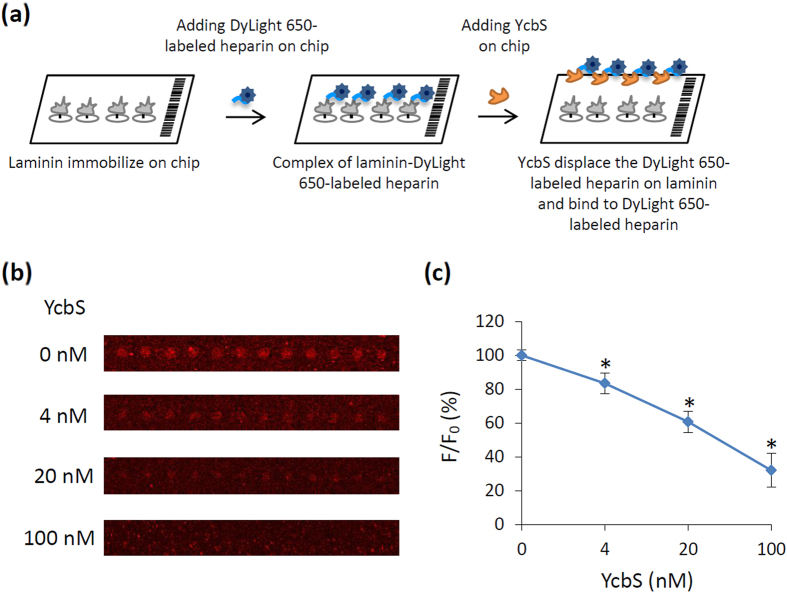
YcbS-induced displacement of the heparin–laminin interaction. (**a**) A schematic diagram of the displacement assays performed after probing DyLight 650-labeled heparin-preincubated laminin chips by using different concentrations of YcbS to measure the capacity of the protein to displace DyLight 650-labeled heparin from binding to laminin. (**b**) The fluorescence signals detected from the DyLight 650-labeled heparin binding to laminin dose-dependently decreased with increasing concentrations of the probed YcbS. (**c**) The plot figure reports the average fluorescence intensity of DyLight 650-labeled heparin binding to laminin in the absence (F0) and presence (F) of YcbS (n = 72). The fluorescence intensities were standardised as percentages. Controls were set to 100%. The asterisks represent the statistically significant difference of the fluorescence intensities of DyLight 650-labeled heparin binding to laminin in increasing concentrations of the probed YcbS compared with the WT control (*p* < 0.05).

Laminins bind to heparin in laminin globular (LG) domains, which play an crucial role in regulating the ECM assembly and stabilise the cell membrane structure^27^. Moreover, the heparin-binding domain of LG domains is a bacterial target. Interference with the laminin–heparin interaction by high-affinity heparin-binding pathogens might destabilise the cell membrane structure, possibly resulting in the loss of appropriate signals and a significant disruption of the cell function^28^,^29^. The present findings revealed YcbS–heparin interactions, which involve bacterial invasions; therefore, we hypothesised that YcbS targets the heparin–laminin interaction. To test this hypothesis, we performed displacement assays to characterise YcbS-mediated heparin–laminin binding through a chip assay approach. Protein chips containing printed laminin were preincubated with DyLight 650-labeled heparin for conducting the binding reaction. After several washes, the displacement assay was performed through further incubation of the chips with different concentrations of purified YcbS (Fig. 5a). The results revealed that YcbS dose-dependently reduces the fluorescence signals of DyLight 650-labeled heparin bound to laminin (Fig. 5b,c). Bovine serum albumin (BSA) was used as a negative control. This result indicated that YcbS targets heparin–laminin interactions by displacing heparin from binding to laminin. This indicated that YcbS binds to the laminin-binding site of heparin and potentially disrupts the host ECM structure, suggesting that YcbS is crucial for bacterial invasion.

## Discussion

We conducted a systematic proteome-wide screening of the *E. coli* protein interactome of four typical mammalian GAGs. Hundreds of proteins were identified as GBPs. We used gene ontology to visualise the enrichment of GBPs in cellular component, biological process, and molecular function. We observed many terms related to cytoplasm were enriched in cellular component (Supplementary Fig. S5a). Analysis in biological processes further revealed several enrichments regarding metabolism (Supplementary Fig. S5b). This result indicated that most of the GBPs were located intracellularly and has functions involving bacterial metabolism, which might reflect interactions of GBPs that contribute to the biosynthesis of the bacterial capsule or other bacterial virulent components with a composition similar to that of mammalian GAGs^30^,^31^. In addition, the molecular function of GBP revealed nucleic acid-binding proteins as the most dominant protein class (Supplementary Fig. S5c). This result was expected because many nucleic acid-binding proteins tend to bind to negatively charged molecules, such as DNA and RNA, and sulphated GAGs^32^. Among the four GAGs, heparin bound to most identified GAG hits, reflecting that heparin is the most negatively charged biological molecule that has been identified^33^,^34^,^35^. As previously described, heparin provides the perfect surface for the attachment of both Gram-positive and -negative bacteria through binding to positively charged proteins on the bacterial surface^36^.

In this study, we focused on three outer membrane GBPs to investigate the implications of these molecules on *E. coli* adherence to the host cells. Invasion assays revealed that YcbS not only binds to the surface of mammalian cells but also plays a crucial role in the invasion of host cells. We also observed that not only *ycbS* but also nearly all the components of the *ycb* operon (*ycbQ*, *ycbR*, *ycbS*, *ycbT*, *ycbU*, and *ycbV*) are critical for bacterial invasion.

*ycb* is a cryptic operon system in *E. coli* K12, producing an extracellular fimbrial-like structure that is crucial for forming biofilms^26^. Therefore, the regulation of this fimbrial-like structure by the *ycb* operon likely contributes to the bacterial attachment to the host cell surface. YcbQ, another component of the *ycb* operon, also binds to laminin and plays a major role in bacterial adherence to the host cells^37^. Therefore, the results revealing YcbS–heparin binding extend the current understanding of the functional roles of the *ycb* operon in *E. coli* infection. In addition, YcbQ and YcbS are two outer membrane components of the fimbrial structure produced by the *ycb* operon^38^, suggesting that these two proteins act as bacterial adhesins and establish a direct interaction with the host cell surface. Considering the discrepancy in the binding preference of YcbS and YcbQ to host ECM components, whether these two proteins function synergistically or alternatively bind to diverse host cell surfaces should be determined. The displacement assay revealed that YcbS displaces heparin from binding to laminin, indicating that YcbS probably contributes to bacterial infection by disrupting the heparin–laminin binding on the host cellular surface. Notably, the heparin–laminin interaction is a pivotal aspect of mammalian ECM organisation, contributing to a series of interactions that result in tight adherence, subsequent internalisation, and potential downstream inflammatory responses that are characteristic of infections^39^. Therefore, YcbS and YcbQ might act cooperatively to facilitate bacterial infections.

In addition to the finding of a novel role of YcbS, our chip assay revealed hundreds of proteins interacted with GAGs. Despite their biological relevance owing to GAG binding, the potential use of GBPs is crucial for the development of disease therapeutics, not only aimed at infection-related diseases but also at cancer treatment. For example, GBPs would be useful for developing biologically active GAG-binding peptides, thus demonstrating an alternative drug therapy against cancers^40^.

In conclusion, this is the first study to systematically screen four typical mammalian GAGs, providing a list of GBPs that are potentially crucial for both further molecular studies and the development of novel therapeutic treatments targeting interactions with cellular GAGs. Further analyses of the outer membrane proteins revealed a role of the *ycb* operon in *E. coli* invasion.

## Methods

### Bacterial strains, cell culture, and chemicals

Single gene knockout mutants of *E. coli* K12 were obtained from the Keio collection^41^; these mutants were designed to create in-frame deletions upon the elimination of the resistance cassette. The open reading frame (ORF) clones of *E. coli* K12 were obtained from the pCA24N-based overexpression ASKA library^42^. Each clone encodes a protein of a predicted ORF attached to His under the control of an IPTG-inducible promoter. Kanamycin (50 μg/mL) was used for preculturing the Keio knockout mutants. The ASKA ORF clone was maintained by adding chloramphenicol (30 μg/mL).

The human ileocecal epithelial cell line HCT-8 (American Type Culture Collection, CCL-244) was maintained in a complete medium composed of an RPMI-1640 medium (Sigma–Aldrich) supplemented with 1 mM sodium pyruvate (Sigma–Aldrich) and 10% (v/v) heat-inactivated horse serum (Invitrogen) at 37 °C under 5% CO_2_ and 95% humidity. The cells were subcultured twice a week at a ratio of 1:3 to 1:8.

The GAGs (Sigma–Aldrich; range, 10–15 KDa) were heparin, HS, CSB, and CSC, and these molecules were used for proteome microarray screening.

### Fabrication of the *E. coli* proteome chips

We constructed a proteome chip comprising approximately 4300 individual purified full-length *E. coli* proteins, as previously reported^12^. *E. coli* clones harbouring vectors expressing 6× His-tagged proteins^42^ were inoculated in 96-deep well plates (Nunc). Expression was subsequently induced using IPTG for 3.5 h; the clones were pelleted and stored at −80 °C overnight.

For protein purification, the thawed cell pellets were resuspended in a lysis buffer composed of 50 mM NaH_2_PO_4_, 300 mM NaCl, 30 mM imidazole, CelLyticB, 1 mg/mL lysozyme, 50 units/mL benzonase, a proteinase inhibitor mixture, 1 mM phenylmethanesulfonyl fluoride, and Ni-NTA Superflow resins (Qiagen). Following a 2.5-h incubation on a plate shaker at 4 °C, the mixtures were transferred into 96-well filter plates (Nunc) and subsequently washed with wash buffer I (50 mM NaH_2_PO_4_, 300 mM NaCl, 20% glycerol, 20 mM imidazole, and 0.1% Tween 20, pH 8.0) and wash buffer II (50 mM NaH_2_PO_4_, 150 mM NaCl, 30% glycerol, 30 mM imidazole, and 0.1% Tween 20, pH 8.0) and subsequently eluted with an elution buffer (50 mM NaH_2_PO_4_, 150 mM NaCl, 30% glycerol, 300 mM imidazole, and 0.1% Tween 20, pH 7.5). The purified proteins were printed in duplicate on Biao aldehyde-coated slides by using ChipWriter Pro (Bio-Rad) and stored at −80 °C until further use.

### DyLight 650 labelling of GAGs

The GAGs were chemically labeled with DyLight 650 (Thermo Fisher Scientific). A mixture of 0.5 mM of each GAG was dissolved in 50 mM borate buffer, and 10 mg/mL of a DyLight 650 NHS ester (molar ratio, 1:20) was incubated at 4 °C for 2 h. The reaction mixture was subsequently incubated with a 10-fold molar excess of Tris-HCl buffer (pH 8.5) to quench the unreacted NHS group and stored at −80 °C until further use.

### Probing the *E. coli* proteome chips with GAGs

Each *E. coli* proteome chip was blocked with 1% BSA to reduce nonspecific binding. Approximately 0.5 μM DyLight 650-labeled heparin, HS, CSB, and CSC in Tris-buffered saline (TBS) with 0.05% Tween 20 and 1% BSA were individually probed with the chip at room temperature for 1 h. Following incubation, the chips were sequentially washed with TBS with 0.05% Tween 20 and distilled water for 5 min to remove excess unbound GAGs. The chips were dried through centrifugation at 201 × *g* and subsequently scanned using a LuxScan-10 K/A array scanner (CapitalBio). The binding signals were acquired and analysed using GenePix Pro 6.0 software. Each GAG-binding experiment was conducted in three separate *E. coli* proteome chips.

### Bioinformatics analysis

GBPs were selected on the basis of a local cutoff, defined as 2 SDs above the regional signal mean (protein spot area, 9 × 9) of the specific protein spot. ProCAT^43^ was used for signal normalisation. All identified GBPs were mapped into functional groups by using the KEGG database^44^,^45^. The consensus motif was searched using GLAM2. The identified GBP sequences were converted to the FASTA format and analysed using GLAM2 to survey consensus motifs^46^. The GLAM2 parameters were set as the default, and the resulting motifs were searched through the entire *E. coli* proteome by using GLAM2SCAN.

### Quantitative real-time reverse transcription PCR (QPCR)

The total cellular RNA was extracted using the MagNA Pure Compact System (Roche). The quantity of RNA was determined using a NanoDrop ND-1000 spectrophotometer (Thermo Fisher Scientific). RNA samples were reverse-transcribed for 120 min at 37 °C with the High Capacity cDNA Reverse Transcription Kit according to the manufacturer’s instructions (Applied Biosystems). QPCR was performed using the 2 × Power SYBR Green PCR Master Mix (Applied Biosystems) and 200 nM forward and reverse primers under the following conditions: 10 min at 95 °C, 40 cycles of 15 s at 95 °C, and 1 min at 60 °C. The used primer sequences are as follows: forward 5′-GAACAGCACCGGGCTGAA-3′ and reverse 5′-TAACGACGCCGCATCAAGT-3′ for *ycbS* and forward 5′-ATACCGCATAACGTCGCAAGA-3′ and reverse 5′-GTGAGCCGTTACCCCACCTA-3′ for 16S rRNA. Each assay was run in triplicate on an Applied Biosystems 7300 Real-Time PCR system (Applied Biosystems), and expression fold changes were derived using the comparative CT method. Moreover, 16S rRNA was used as the reference gene to normalise specific gene expression in each sample.

### Complementation of *ycbS* in the Keio *E. coli* mutants

The *ycbS*-lacking *E. coli* mutant was obtained from the Keio collection^41^. To complement the Keio *ycbS* mutants, the plasmid pCA24N–ycbS harbouring *ycbS* was obtained from the ASKA clone library^42^ and used to transform the *ycbS* mutant, as previously described^47^.

### Quantitative cell invasion assays

To determine the bacterial invasion into epithelial cells, the spread plate bacterial colonies were first preinoculated in 3 mL of a fresh Luria–Bertani (LB) medium with shaking overnight at 37 °C and 30 × g. Approximately 1 mL of the bacterial cultures were then inoculated into 20 mL of a LB medium and incubated with shaking at 37 °C and 30 × g until they reached the midlogarithmic phase (optical density at 600, 1.0). For the cell invasion assays, 2 × 10^7^ CFU/mL of bacterial cells were added to confluent HCT-8 cells (approximately 2.3 × 10^5^ cells in a 12-well tissue culture plate) in a complete medium composed of an RPMI-1640 medium supplemented with 1 mM sodium pyruvate and 10% heat-inactivated horse serum. The plate was incubated at 37 °C under 5% CO_2_ and 95% humidity for 3 h. The infected epithelial cells were subsequently incubated with 100 μg/mL of gentamycin in a complete medium after five washes with an RPMI-1640 medium (Sigma–Aldrich). Furthermore, the cells were incubated for an additional 1 h at 37 °C under 5% CO_2_ and 95% humidity to kill the extracellular bacteria, as previously reported^25^. Next, the HCT-8 cells were washed five times with an RPMI-1640 medium and disrupted with 0.1% Triton X-100, and the released bacterial cells were quantified through the spread plate method. The cell invasion efficiency was determined as the percentage of bacterial cells recovered from the five biological repeat wells compared with 100% efficiency in the cells infected with their isogenic parental strain (WT control).

In experiments in which *ycbS* was supplied using the pCA24N plasmid, the spread plate bacterial colonies were first preinoculated in 3 mL of a fresh LB medium with shaking overnight at 37 °C and 30 × g. Approximately 1 mL of the bacterial cultures were subsequently transferred to 20 mL of a fresh LB medium and incubated with shaking at 37 °C and 30 × g until they reached an optical density at 600 of 0.7. Next, different concentrations of IPTG was added to the bacterial cultures, including the WT control, and the cultures were incubated at 37 °C and 30 × g for 3 h^48^ to ensure YcbS expression from the inoculated bacteria during the invasion assay.

### Blocking assays

The purified YcbS (10, 50, and 100 nM) was incubated with the HCT-8 cells at 37 °C for 1 h. The cells were subsequently washed twice with an RPMI-1640 medium (Sigma–Aldrich) and subjected to cell invasion assays, as described in the previous section.

### Kd determination by using SPR

A customised SPR biosensor^49^ built according to wavelength interrogation with a five-channel Teflon flow cell was used to monitor the recognition interaction between heparin and YcbS. The bare gold surface of an SPR chip was first thoroughly washed with deionised water and absolute ethanol, dried under argon, and immediately immersed in a mixture of 0.5 mM 11-amino-1-undecanethiol hydrochloride and 1.5 mM 6-mercapto-1-hexanol in absolute ethanol. The chip surface was subsequently immersed in 12.5% glutaraldehyde for 1 h at room temperature. Furthermore, YcbS (1 μM) was covalently immobilised on the activated surface overnight; the sensor chip surface was cleaned with pure water and dried using nitrogen before use. To obtain interaction kinetic measurements, the SPR was stabilised in TBS for 15 min. The indicated concentration of heparin was injected for 15 min (flow rate = 18.2 μL/min) and subsequently dissociated for 15 min. The affinity interactions between immobilised YcbS and heparin in the solution were determined, as previously reported^50^.

### Flow cytometry

Flow cytometry was used to validate YcbS binding to the HCT-8 cells. YcbS was purified and verified through sodium dodecyl sulphate (SDS) polyacrylamide gel electrophoresis (PAGE). The HCT-8 cells were detached using 10 mM ethylenediaminetetraacetic acid–phosphate-buffered saline (PBS) and incubated with 100 nM YcbS at 4 °C for 1 h. The cells were subsequently washed twice with PBS containing 0.1% BSA at 157 × g for 5 min to remove the unbound proteins. The cells were further stained with DyLight 650-tagged anti-His antibody at 4 °C for 30 min to capture the cell–YcbS complex. After washing through centrifugation at 157 × g for 5 min, 5000 cells were collected from each sample and analysed using the Muse Cell Analyser (Merck Millipore). The processed data were acquired as screenshots, and the text size was edited for clarity. All the results were compared with untreated cells stained with anti-His antibody in triplicate.

### YcbS-induced displacement of heparin–laminin binding

To evaluate YcbS displacement in heparin–laminin binding, the protein chips were printed using 150 μg/mL of laminin (EMD Millipore) and immobilised at 4 °C for 12 h. The chips were subsequently incubated with 1.34 μM of DyLight 650-labeled heparin for 1 h at room temperature to facilitate laminin binding to DyLight 650-labeled heparin. After being washed with TBS containing 0.05% Tween 20, the chips were further incubated with three concentrations (4, 20, and 100 nM) of purified YcbS and rinsed with distilled water following incubation for 20 min. The remaining DyLight 650-labeled heparin signal was obtained using a LuxScan laser scanner (CapitalBio).

### Statistical analyses

The cumulative hypergeometric distribution (or Fisher exact test, referred to as the hypergeometric test) was used to score the motif enrichment. Invasion assay results were presented as mean ± standard error; the experimental and control groups were compared through the two-tailed Student t test.

## Additional Information

**How to cite this article**: Hsiao, F. S.-H. *et al*. Systematic protein interactome analysis of glycosaminoglycans revealed YcbS as a novel bacterial virulence factor. *Sci. Rep.* **6**, 28425; doi: 10.1038/srep28425 (2016).

## Supplementary Material

Supplementary Information

Supplementary Dataset 1
