# Supplementary material for: Systematic protein interactome analysis of glycosaminoglycans revealed YcbS as a novel bacterial virulence factor
Source: Sci Rep. 2016 Jun 21;6:28425. doi: 10.1038/srep28425 (PMC4914927; doi:10.1038/srep28425)
Supplement: Supplementary Information [file srep28425-s1.doc]

**Supplementary information**

**Systematic protein interactome analysis of glycosaminoglycans revealed YcbS as a novel bacterial virulence factor**

Felix Shih-Hsiang Hsiao^1,2,+^, FX Reymond Sutandy^1,2,+^, Guan-Da Syu^1,2^, Yi-Wen Chen^1,2^, Jun-Mu Lin^1,2^, and Chien-Sheng Chen^1,2,*^

^1^ Graduate Institute of Systems Biology and Bioinformatics, National Central University, Jongli District, Taoyuan City 32001, Taiwan

^2^ Department of Biomedical Science and Engineering, National Central University, Jongli District, Taoyuan City 32001, Taiwan

^*^ corresponding author: cchen103@gmail.com

^+^ these authors contributed equally to this work

Supplementary Figure S1

**
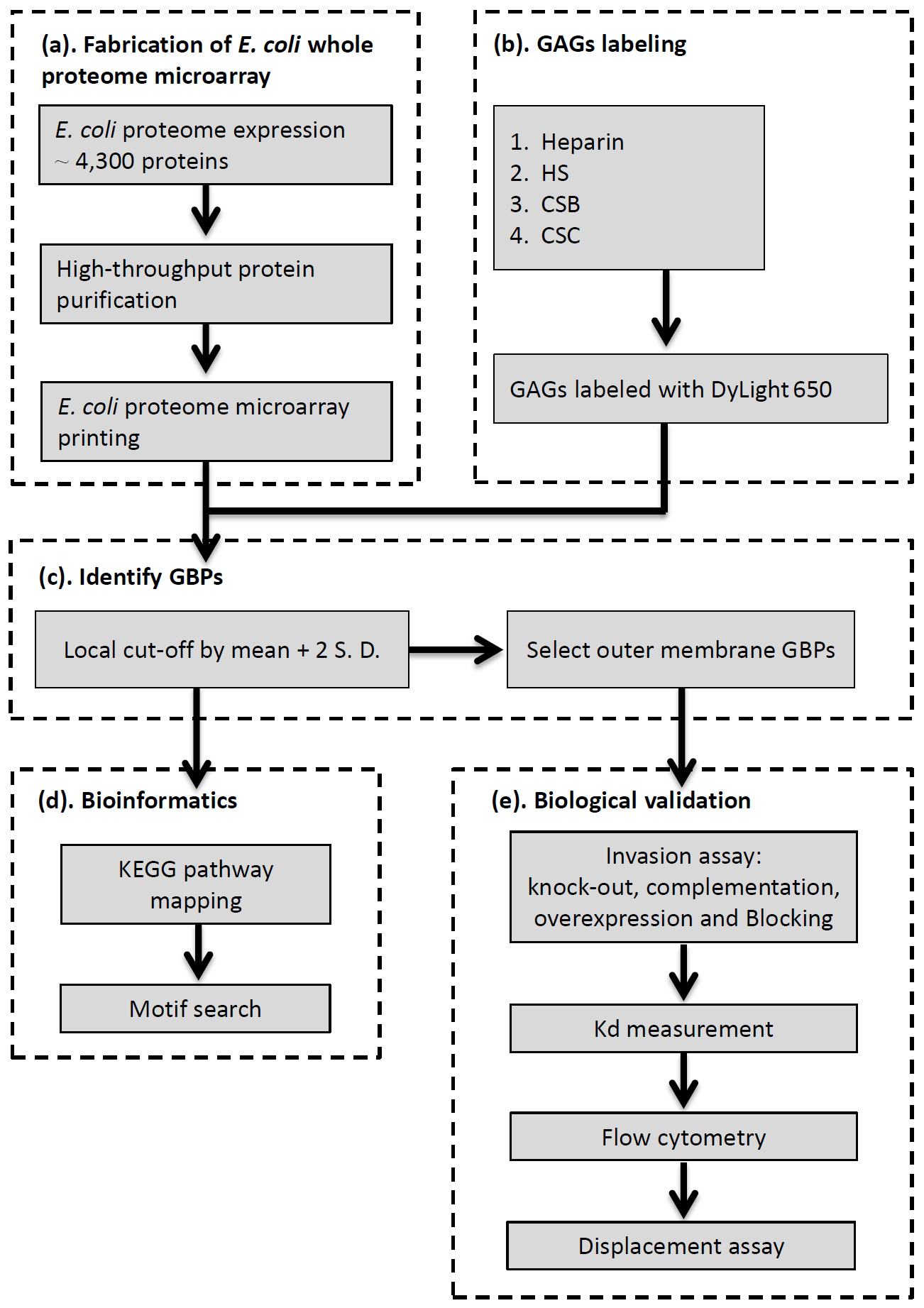
**

**Supplementary Figure S1: Overall strategy for identifying GBPs by using the *E. coli* proteome chip**

To profile the GAG–protein interactome, we probed *E. coli* proteome chips with the labelled GAGs. (a) To fabricate the *E. coli* whole proteome chip, we expressed and purified approximately 4300 *E. coli* proteins before printing in a cold room. (b) The GAG molecules were labelled with DyLight 650. These labelled GAGs were subsequently probed using *E. coli* proteome chips. (c) GBPs were identified using a local cutoff value of mean + 2 SD of the normalised GAG-binding signals. (d) To determine the biological relevance of the GBPs, KEGG pathway mapping and a motif search were performed. (e) The outer membrane GBPs were selected from all the GBPs; the biological functions of these molecules were validated using *E. coli* invasion assays, including knockout, complementation, overexpression, blocking, Kd measurement, flow cytometry, and displacement assays.

Supplementary Figure S2

**
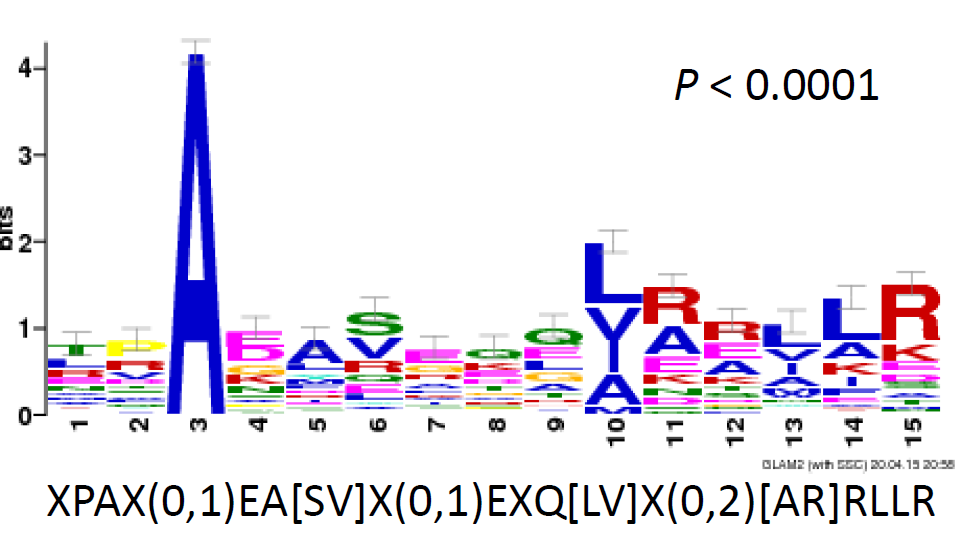
**

**Supplementary Figure S2: Motif search of heparin- and HS-specific GBPs**

The conserved protein sequences (*p* < 0.0001) of heparin- and HS-specific GBPs were observed using the motif-based sequence analysis tool GLAM2. The standard IUPAC one-letter code for amino acids is used in PROSITE (<http://prosite.expasy.org/>). The symbol X is used for a position where any amino acid is accepted, whereas X(0,1) corresponds to none or one (X) amino acid at an indicated position and X(0,2) corresponds to none, one, or two (X) amino acids at an indicated position.

Supplementary Figure S3


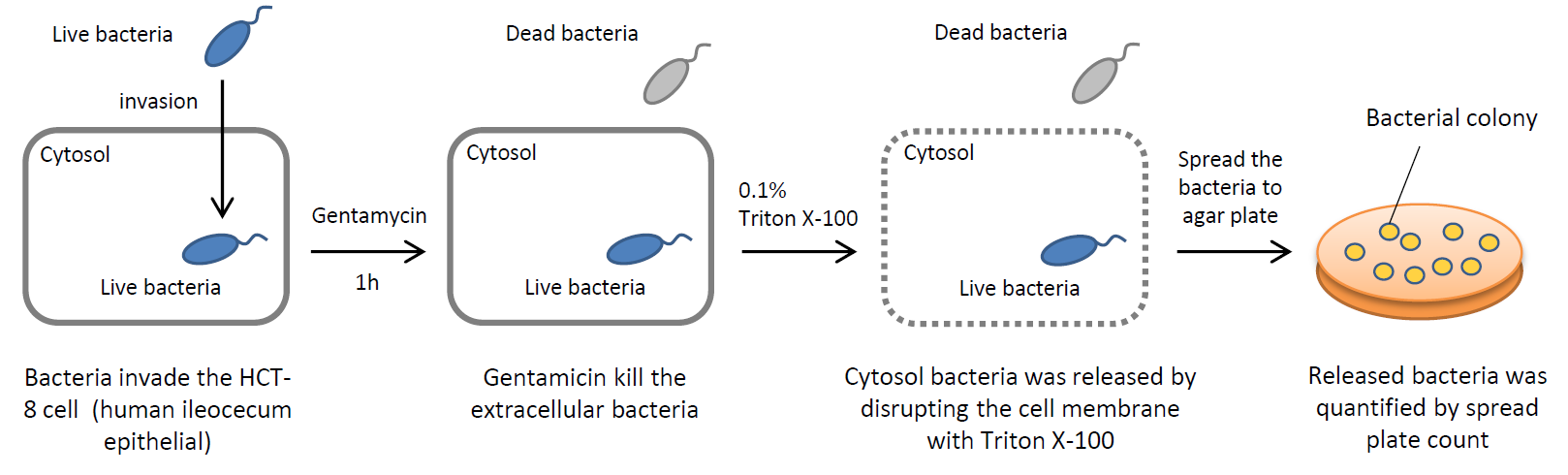


**Supplementary Figure S3: Schematic diagram of *E. coli* invasion of HCT-8 cells**

The bacteria were added to the human ileocecal epithelial cell line HCT-8 for invasion. The HCT-8 cells were subsequently incubated with gentamycin to kill extracellular noninvading bacteria. The cytosol-invading bacteria were released after the HCT-8 cells were disrupted with 0.1% Triton X-100 and quantified through the spread plate method.

Supplementary Figure S4


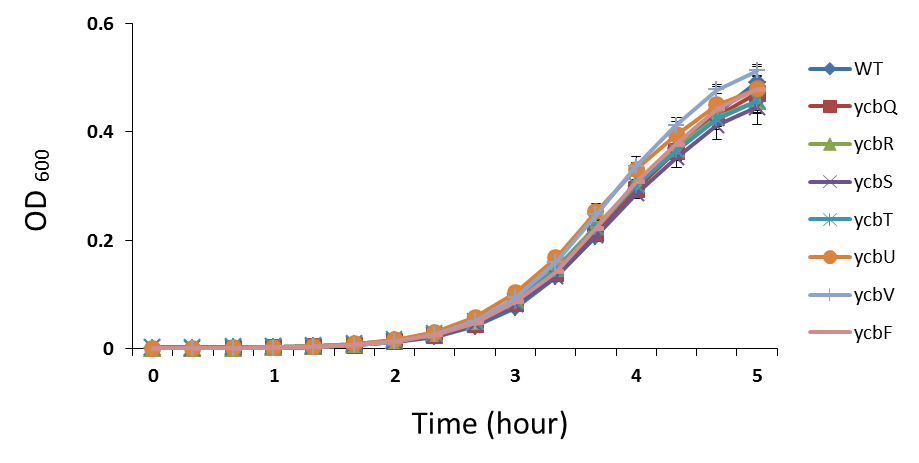


**Supplementary Figure S4: *ycbQRSTUVF*-knockout *E. coli* mutants exhibit similar growth kinetics in culture**

The *E. coli* mutants were first inoculated into the LB medium and incubated at 37 °C with shaking for 16 h. Following the incubation, the culture was diluted to approximately 10^7^ CFU/ml in a 96-well plate. The diluted *E. coli* was incubated at 37 °C, and the growth curves were observed by measuring the OD600 value every 20 min for 5 h by using a microplate reader (Synergy 2, BioTekH).

Supplementary Figure S5

**
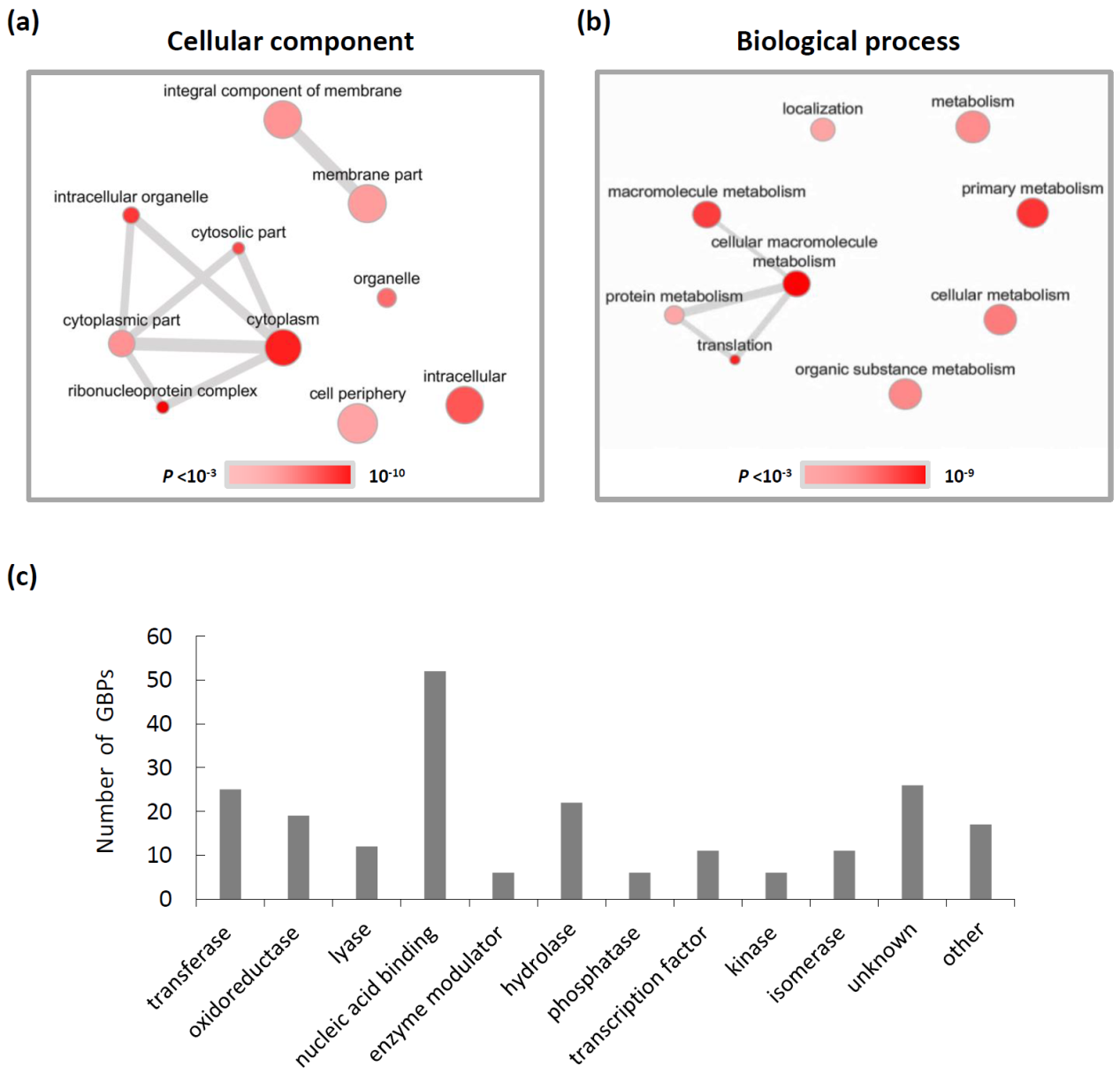
**

**Supplementary Figure S5: Bioinformatics analyses of GBPs**

REVIGO and Cytoscape were used to represent the significantly enriched gene ontology terms regarding the (a) cellular component and (b) biological processes of all the identified GBPs (*p* < 0.001). (c) The GBPs were classified into different protein classes by using PANTHER. Nucleic acid-binding proteins are the most dominant class of GAGs, followed by several classes of metabolic proteins.
